# Clinical differential factors in patients with hereditary transthyretin amyloidosis with Val142Ile and Ser43Asn mutations

**DOI:** 10.1186/s13023-024-03496-0

**Published:** 2024-12-20

**Authors:** Sandra Milena Castellar-Leones, Edicson Ruiz-Ospina, Jorge Diaz-Ruiz, Cristian Correa-Arrieta, Xiomara Ruiz-Cortés, Diana Luzuriaga-Carpio, Dario Zambrano-Vera, Jeanneth Cedeño-Quincha, Luis Guerrero-Cepeda, Daniel César-Chávez, Fernando Ortiz-Corredor

**Affiliations:** 1 Department of Physical Medicine and Rehabilitation, Facultad de Medicina, Universidad Nacional de Colombia, Carrera 30 No.45‐03. Edificio 471, Piso 5to, Of. 513‐A, Bogotá, Colombia; 2 Research Center for Physiatry and Electrodiagnostics, (CIFEL), Bogotá, Colombia; 3 Department of Neurogenetic, Neuromuscular and Neurodegenerative Diseases, Biotecnología y Genética (Biotecgen), Bogotá, Colombia; 4 Center of Rehabilitation RecuperAMI, Caquetá, Colombia; 5 Hospital General Manuel Ygnacio Monteros-IESS, Loja, Ecuador; 6 Hospital de Especialidades Carlos Andrade Marín-IESS, Quito, Ecuador; 7 Hospital de Especialidades Eugenio Espejo, Quito, Ecuador; 8 Hospital Teodoro Maldonado Carbo-IESS, Guayaquil, Ecuador

**Keywords:** Hereditary transthyretin amyloidosis, Quantitative sensory testing, Composite Autonomic Symptom Score 31, Neuropathy Impairment Score, Nerve ultrasound, Nerve cross-sectional area

## Abstract

**Background:**

Hereditary transthyretin amyloidosis (hATTR) is a rare autosomal dominant disease with high clinical variability, influenced by both genotype and the geographic origins of carriers. There is a limited understanding of the Val142Ile and Ser43Asn recognised mutations in Ecuador and Colombia. Therefore, the objective of this study is to describe the neurological and functional characteristics of patients with hATTR associated with the Val142Ile and Ser43Asn mutations, as well as to identify possible differentiating factors between the two mutations.

**Methods:**

This cross-sectional, multicenter study included 35 hATTR patients from rehabilitation centers in Ecuador and Colombia. Patients had confirmed Val142Ile or Ser43Asn mutations. Neurological and functional assessments included the Neurological Impairment Scale, Norfolk Quality of Life-Diabetic Neuropathy (QOL-DN), Composite Autonomic Symptom Score-31, and various motor function tests as nine-hole peg test (NHP). Quantitative Sensory Testing (QST) evaluating small fiber function, while ultrasound measured the cross-sectional area (CSA) of peripheral nerves. Statistical analysis employed nonparametric tests and random forest classifiers, using SHAP values to identify differentiating variables.

**Results:**

Val142Ile carriers showed lower performance in the right NHP test and greater sensitivity to cold pain in hand and leg. Ultrasound revealed increased CSA of the median nerve at the elbow and arm and the ulnar nerve at the arm in Val142Ile carriers compared to Ser43Asn carriers. The final random forest model identified the NHP test, Norfolk QOL-DN score, and CSA of the median and ulnar nerves as key discriminating variables.

**Conclusion:**

This study identified significant neurophysiological and ultrasound markers differentiating Val142Ile and Ser43Asn mutations in hATTR-PN patients. Increased nerve CSA and specific motor and sensory impairments highlight the need for comprehensive evaluations to guide diagnosis and treatment.

**Supplementary Information:**

The online version contains supplementary material available at 10.1186/s13023-024-03496-0.

## Introduction

Hereditary transthyretin amyloidosis (hATTR) is a rare autosomal dominant genetic disease associated with mutations in the transthyretin (*TTR*) gene and adult onset [1, 2]. Clinical variability is observed in hATTR, which is associated with genotype, age, mutation penetrance, and geographic origin [1, 3–5]. One of the most common clinical manifestations of hATTR is a progressive peripheral polyneuropathy (hATTR-PN) accompanied by sensory and motor symptoms [6–8]. The recognition and comprehension of these clinical variations are crucial for an accurate and timely diagnosis of the disease onset through clinical assessments [9, 10]. This, in turn, allows for the implementation of an appropriate and personalized therapeutic approach [11].

Over 120 mutations of the *TTR* gene have been identified, with the most common being the Val50Met mutation, which is associated with multi-organ involvement and early-onset neuropathy [1, 10, 12]. Additionally, certain mutations have been associated to specific geographic origin. For instance, the Phe64Leu mutation, which is the most prevalent in Italy [4], is associated with lower amyloid deposits in the nerve biosynthesis and a slower disease progression compared to other mutations [6, 13]. Other geographically associated mutations, such as Val30Met or Val122, have also been described [1, 4, 8]. However, descriptions of less frequent mutations or present in developing regions, such as Latin America, are rather scarce.

The international literature on the prevalence and clinical characteristics of hATTR-PN in Latin America is notably scarce. However, some local studies suggest a marked prevalence of the Val142Ile mutation in Colombia [14] and the Ser43Asn mutation in Ecuador [15]. The Val142Ile mutation, more common in individuals of African descent, is predominantly associeted with development of heart disease [16]. Nevertheless, it has been observed that carriers of this mutation often request medical attention for neurological symptoms consistent with hATTR-PN [17]. Conversely, the Ser43Asn mutation, found in both Italy and Ecuador, where it may be endemic [15], is identified as a rare mutation with potential isolated cardiac phenotypes [18, 19]. Additionally, it has been associated with polyneuropathy symptomatology [18]. Despite these observations, detailed neurological and functional information on these two mutations remains limited due to the inherent challenges of studying rare diseases. Therefore, this study aims to describe the neurological and functional characteristics of patients with hATTR-PN associated with the Val142Ile and Ser43Asn mutation, as well as to identify possible differentiating factors between the two mutations.

## Methods

### Study population

This cross-sectional and multicenter study was conducted between August 2023 and May 2024 with patients who were admitted to the Hospital de Especialidades Carlos Andrade Marín-IESS, Hospital de Especialidades Eugenio Espejo, Hospital Teodoro Maldonado Carbo-IESS and Hospital General Manuel Ygnacio Monteros-IESS located in Quito, Guayaquil and Loja Ecuador, as well as at the RecuperAmi rehabilitation center in Caquetá, Colombia and the Centro de Investigación en Fisiatría y Electrodiagnóstico (CIFEL) in Bogotá, Colombia. All patients were informed of the study, and their consent was obtained for the use of their data. This study was approved by the local ethics committee of CIFEL (file number 20240605–001). The CIFEL research team also supervised and conducted analysis of this study. All patients with a confirmed diagnosis of hATTR over the age of 18 were included. Patients with missing data on functional evaluations or ultrasound were excluded.

#### Genetic confirmation of hereditary transthyretin amyloidosis

The patients included in this study presented with Val142Ile and Ser43Asn mutations, originating from Colombia and Ecuador, respectively. Prior to their inclusion in the study, the diagnosis genetic analysis and confirmation of diagnosis were performed by external services. Clinical confirmation was conducted by physiatrists and neurologists at the respective health centers.

#### Measures

Each participant was evaluated at the time of consultation by physiatrists, neurologists, and rehabilitation professionals. Measures of global function and well-being included the Neurological Impairment Scale (NSI) [20], Norfolk Quality of Life-Diabetic Neuropathy (QOL-DN) [21], Composite Autonomic Symptom Score-31 (Compass-31) [22]. To assess fine and gross motor functions, the following tests were employed: the nine-hole peg test (NHP), the timed up and go test (TUG), the sit and stand 5 times test (SST5), and the monopodal static balance test (MSB) [23–25].

The sensory aspects were measured with Quantitative Sensory Testing (QST), assessing hyposensitivity, hypersensitive or normo-sensitivity in four types of sensation: warm sensation, cold sensation, hot pain and cold pain through method of limits [26]. This evaluation was performed with a 3 × 3 thermode at a base temperature of 32 °C, decreasing at a rate of 1 °C/s to 10° and increased to 50 °C, rate 0.1–4 °C/s and automatic tigger mode. The anatomical sites where QST was performed were dorsum of the hand, distal anterior aspect of the leg and dorsum of the foot. Each threshold was represented as the absolute deviation in temperature from the baseline temperature. The device used was the Thermal Sensory Analyzer II (TSA-2001, Medoc Ltd., Ramat Yishai, Israel). Additionally, the Neuropathic Pain Scale (NPS) was employed for sensory testing also [27].

Ultrasound imaging was used to assess the cross-sectional area of the peripheral nerves, including the median nerve at the mid-forearm, elbow, and mid-arm levels; the ulnar nerve at the mid-forearm and mid-arm levels; the fibular nerve at the fibular head; and the tibial nerve at the popliteal fossa and ankle. An ultrasound system SonoScape E2 Pro (SonoScape, Medical Corp, Shenzhen, China) with a 4–16 MHz linear-array probe was used. The cross-sectional area was obtained by maintaining the transducer perpendicular to the surface of the nerve, without putting any pressure on the skin, and estimated by tracing a manual continuous circumference of nerve, excluding the hyperechoic epineural rim. All assessment was carried out with participants in supine position.

Data regarding comorbidities such as cardiac, hepatic, renal, ocular, autonomic, carpal tunnel syndrome or other conditions were collected during a consultation with a physiatrist based on clinical evaluation, patient report and confirmation by medical records. The specificities of the morbidities can be consulted in the supplementary data, Table S1.

### Statistical analysis

#### Descriptive analysis

Given the limited number of participants, the population with the Val142Ile mutation was compared with the population with the Ser43Asn mutation using nonparametric statistical tests. The Wilcoxon–Mann–Whitney test was employed to compare the means of quantitative variables, while the chi-square test with Yates correction or Fisher’s exact test was used to assess the proportion between groups. The effect size was estimated for each comparison using the M and E Tomczak method [28] for continuous variables and Cohen’s W was estimated for categorical variables. The thresholds for effect size categorization followed standard conventions: 0.1, 0.3, and 0.5 for small, medium, and large effects in both continuous variables and categorical variables. The p-values were adjusted using the False Discovery Rate method in order to control for type I error.

#### Main analysis

To identify the variables that differentiate the two mutations, a random forest classifier was calibrated. The nonparametric random forest model was selected for its resilience to outliers, lack of assumptions about the associations about variables association, capacity to handle high dimensionality, ability to quantify the variable importance, and consideration of potential interactions between variables. The interpretation of the model’s variable importance was further enhanced by calculation of the SHapley Additive exPlanations (SHAP) values, which provide a local explanation of how each variable affects individual predictions [29]. See the supplementary methods for more information on estimating SHAP values.

To facilitate the interpretation of the results and reduce the dimensionality of the analysis, four initial models were calibrated, grouping variables such as functional scales, sensory measures, the cross-sectional area (CSA) by ultrasound, and comorbidities. Subsequently, the Shap values of the four models were estimated, and the most contributory variables from each model were selected to calibrate a final model. Both the initial four models and the final model were adjusted for age and sex.

#### Sensitivity analysis

Given the limited sample size and the potential for overfitting bias, a continuous variable and a categorical variable were randomly split between the two groups. These variables were included in each model and in the final model to evaluate the possibility of bias in the identification of differentiating variables.

#### Post-hoc analysis

In consideration of the limited statistical power of our analyses, it is plausible that the observed lack of association of sex and its interaction with nerve CSA, with study mutations may be attributed to error type II. To address this potential issue, a sex-stratified replication analysis was conducted.

All analysis was conducted in R version 4.2.1 and Python 3.9 and “shap 0.46.0”, “effectsize” libraries.

## Results

The study included three women and seven men with hATTR carrying the Val142Ile mutation from Colombia, with a mean age of 42.2 (SD = 18.2). Additionally, it included 11 women and 14 men with hATTR carrying the Ser43Asn mutation from Ecuador with a mean age of 37.76 (SD = 14.7). No significant difference in sex or age distribution was found between the two populations (*p* > 0.08 for both).

Table 1 indicates that individuals with the Val142Ile mutation demonstrated inferior performance on the right NHP test (*p* < 0.019, effect size = 0.40 [Medium]) and exhibited greater sensitivity to cold pain in the hand and leg (*p* < 0.009 for both, effect size = 0.44–0.47 [Medium]) in comparison to Ser43Asn carriers. The CSA of the median nerve at the elbow and arm was found to have increased significantly (*p* = 0.001, effect size = 0.55 [large] for both), as was the CSA of the ulnar nerve at the forearm and elbow (*p* < 0.012 for both, effect sizes = 0.42 [Medium] and 0.55 [large], respectively) in Val142Ile mutation compared with Ser43Asn carriers. Furthermore, the CSA of the tibial nerve at the knee and ankle was observed to be significantly different between Val142Ile carriers and Ser43Asn carriers (*p* <  = 0.020 for both, effect sizes = 0.39–0.40 [medium]). Following the application of the false discovery rate to the *p*-values, only the CSAs of the median nerve at the elbow and arm levels, as well as the ulnar nerve at the arm level, remained significantly associated with mutation type. No other differences were observed in the other tests or in the distribution of comorbidities.

**Table 1:** Clinical and functional characteristics of participants with Val142Ile versus Ser43Asn mutation carriers

Characteristics	Ser43Asn (N = 25)	Val142Ile (N = 10)	*p* value	Adjusted *p* value**	Effect size
Age, mean (SD)	37.76 (14.70)	42.20 (18.23)	0.648	0.759	0.08 (0.01–0.43)
Sex, female	11 (44.0%)	3 (30.0%)	0.445	0.629	0.13 (0.00–0.46)
Neurological impairment scale	4.56 (10.50)	1.20 (3.79)	0.360	0.547	0.15 (0.01–0.38)
Timed up and go test	9.67 (1.93)	9.73 (1.62)	0.729	0.792	0.06 (0.01–0.37)
5-time Sit down-to-Stand up test	9.36 (2.64)	9.97 (2.07)	0.243	0.481	0.20 (0.01–0.48)
Nine-hole peg test, right	19.31 (3.67)	21.34 (2.91)	0.019*	0.091	0.40 (0.10–0.64)
Nine-hole peg test, left	20.79 (3.97)	19.88 (3.27)	0.439	0.629	0.14 (0.01–0.47)
Norfolk quality of life-diabetic neuropathy	12.40 (17.59)	16.00 (15.60)	0.124	0.325	0.26 (0.02–0.56)
Composite Autonomic Symptom Score-31	10.12 (11.60)	13.80 (8.97)	0.216	0.443	0.21 (0.01–0.49)
Monopodal static balance test, right	46.72 (20.60)	48.15 (18.96)	0.966	0.966	0.01 (0.00–0.36)
Monopodal static balance test, left	43.80 (22.52)	45.22 (22.48)	0.934	0.957	0.01 (0.01–0.38)
NPS	11.52 (18.14)	17.60 (15.27)	0.157	0.379	0.24 (0.01–0.54)
*Quantitative sensory testing, hyposensitivity*					
Cold detection hand	6 (24.0%)	1 (10.0%)	0.350	0.547	0.16 (0.00–0.49)
Cold detection leg	7 (28.0%)	2 (20.0%)	0.625	0.759	0.08 (0.00–0.41)
Cold detection foot	6 (24.0%)	0 (0.0%)	0.089	0.325	0.29 (0.00–0.62)
Warm detection hand	9 (36.0%)	4 (40.0%)	0.825	0.867	0.04 (0.00–0.34)
Warm detection leg	4 (16.0%)	3 (30.0%)	0.350	0.547	0.16 (0.00–0.49)
Warm detection foot	2 (8.0%)	0 (0.0%)	0.357	0.547	0.16 (0.00–0.49)
Cold pain hand	4 (16.0%)	6 (60.0%)	0.009*	0.074	0.44 (0.11–0.77)
Cold pain leg	2 (8.0%)	5 (50.0%)	0.005*	0.051	0.47 (0.14–0.81)
Cold pain foot	2 (8.0%)	2 (20.0%)	0.313	0.547	0.17 (0.00–0.50)
Heat pain hand	3 (12.0%)	2 (20.0%)	0.541	0.693	0.10 (0.00–0.43)
Heat pain leg	6 (24.0%)	3 (30.0%)	0.714	0.792	0.06 (0.00–0.38)
Heat pain foot	3 (12.0%)	0 (0.0%)	0.252	0.481	0.19 (0.00–0.52)
*Cross-sectional area of the peripheral nerves by ultrasound (mm*^*2*^*)*					
Median nerve, forearm	7.32 (1.14)	6.60 (1.26)	0.122	0.325	0.26 (0.02–0.56)
Median nerve, elbow	12.04 (2.56)	16.90 (4.31)	0.001*	0.014*	0.55 (0.32–0.73)
Median nerve, arm	13.88 (4.65)	21.70 (5.89)	0.001*	0.014*	0.55 (0.27–0.76)
Ulnar nerve, forearm	7.20 (1.85)	9.80 (3.12)	0.012*	0.082	0.42 (0.08–0.69)
Ulnar nerve, arm	10.20 (3.51)	20.00 (8.81)	0.001*	0.014*	0.55 (0.29–0.73)
Fibular nerve, Fibular head	16.32 (4.10)	21.80 (9.64)	0.124	0.325	0.26 (0.02–0.61)
Tibial nerve, knee	31.88 (14.98)	41.90 (10.57)	0.020*	0.091	0.39 (0.12–0.63)
Tibial nerve, ankle	17.52 (4.31)	23.30 (6.22)	0.017*	0.091	0.40 (0.07–0.68)
*Comorbidities*					
Carpal tunnel syndrome, yes	4 (16.0%)	4 (40.0%)	0.127	0.325	0.26 (0.00–0.59)
Cardiacs, yes	11 (44.0%)	2 (20.0%)	0.184	0.397	0.22 (0.00–0.56)
Hepatics, yes	1 (4.0%)	0 (0.0%)	0.521	0.689	0.11 (0.00–0.44)
Renal, yes	2 (8.0%)	3 (30.0%)	0.093	0.325	0.28 (0.00–0.62)
Ocular, yes	4 (16.0%)	1 (10.0%)	0.647	0.759	0.08 (0.00–0.4)
Otros, yes	5 (20.0%)	0 (0.0%)	0.127	0.325	0.26 (0.00–0.59)
Autonomics, yes	12 (48.0%)	6 (60.0%)	0.521	0.689	0.11 (0.00–0.44)
Number of comorbidities, yes	1.40 (1.41)	1.20 (1.23)	0.820	0.867	0.04 (0.01–0.37)

^*^Significant. **Adjustment of *p* value with False Discovery Rate

**Fig. 1:**
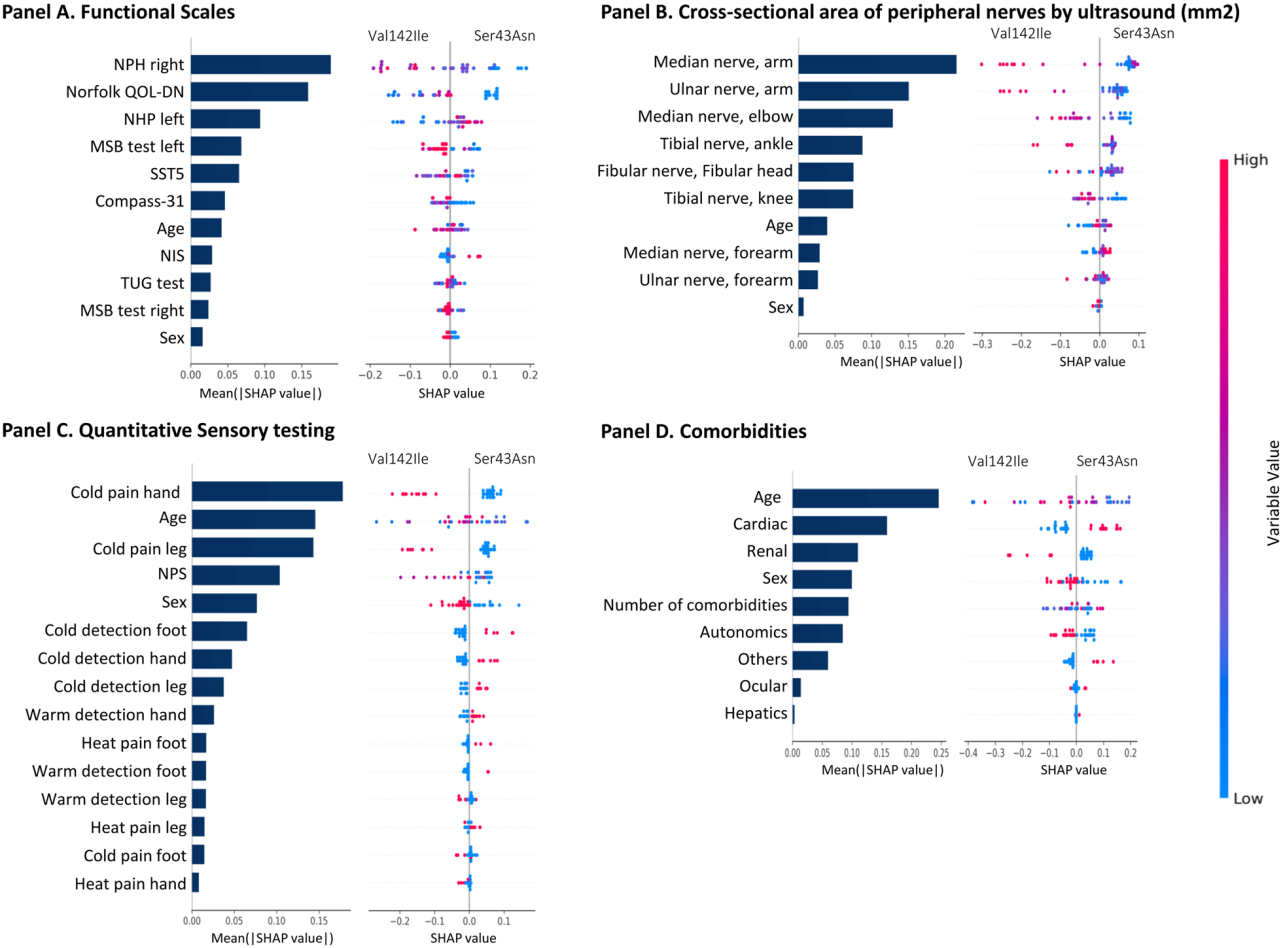
Importance of variables in the predicting Val142Ile or Ser43Asn mutations using random forest and SHAP. In each panel, models were adjusted between variables of the same domain, in addition to sex and age. In each panel, the left side represents the relative importance of the variable, expressed in terms of the Mean(|Shap value|). The right side of each point represents the SHAP value (contribution to the prediction), the further away from 0, the higher the contribution is considered. The colour represents the value of the variable to each prediction, towards red the value of a continuous variable is higher, in a categorical variable it is the value assigned to class 1. For sex: female = 0, male = 1. In the quantitative sensory test: Hyposensitivity = 1, Normal = 0. In comorbidities: No = 0, Yes = 1. SHAP: SHapley Additive exPlanations, NSI: Neurological Impairment Scale, TUG: Timed Up and go test, SST5:5-time Sit down-to-Stand up test, NHP: Nine-hole peg test, QOL-DN: Quality of Life-Diabetic Neuropathy, MSB: Monopodal static balance, Compass-31: Composite Autonomic Symptom Score-31, NPS: Neuropathic Pain Scale

Figure 1 shows the main results of the calibration of the four random forest classifiers as a multivariate model, together with the SHAP values of each model. The right side of each panel shows the mean absolute SHAP values of each variable, indicating which variables influence the predictions of each mutation the most (see Table S2). The right side provides detailed information on how each variable influences individual predictions, with colours representing whether high (red) or low (blue) values of the features drive the prediction. The models allowed us to determine that within the functional assessment, the NHP test performed with the right upper limb is the variable that best discriminates between the two groups (mean(|SHAP value|) = 0.19; Fig. 1, panel A). This is followed by the NorfolkQOL-DN (mean(|SHAP value|) = 0.16), and the NHP with the left upper limb (mean(|SHAP value|) = 0.09). Individuals with longer times to complete the right NHP (red and purple dots) were more likely to belong to the Val142Ile mutation group, while the left NHP showed a less consistent trend, being more likely to belong to the Ser43Arg (Fig. 1, panel A, right side). Individuals exhibiting high values on the Norfolk QOL-DN score (red dots) were more likely to belong to the Val142Ile group than to the Ser43Asn group.

The cross-sectional area of the median and ulnar nerves at arm level and of the median nerve at elbow level proved to be the most discriminating variables between the two groups with Val142Ile and Ser43Arn mutation (mean(|SHAP value|) = 0.21, 0.15 and 0.13, respectively, Fig. 1, panel B, left side). Individuals with a larger cross-sectional area (red dots) were more likely to belong to the Val142Ile group than to the Ser43Arn group, except for 4 subjects (Fig. 1, panel B right side). Model with quantitative sensory testing variables showed that the detection of cold pain with the hand (mean(|SHAP value|) = 0.18) was the sole discriminating variable over age ((mean(|SHAP value|) = 0.14); Fig. 1, panel C left side). The detection of cold pain in the hand contributed to the prediction of the model to a similar extent as age (mean(|SHAP value|) = 0.14 for both). Individuals with cardiac morbidity were more likely to be in the Ser43Asn group (mean(|SHAP value|) = 0.16, Fig. 1, panel D right side) and those with renal morbidity were more likely to be in the Val142Ile group (mean(|SHAP value|) = 0.11). however, neither morbidity demonstrated a greater predictive contribution than age (mean(|SHAP value|) = 0.25).

**Fig. 2:**
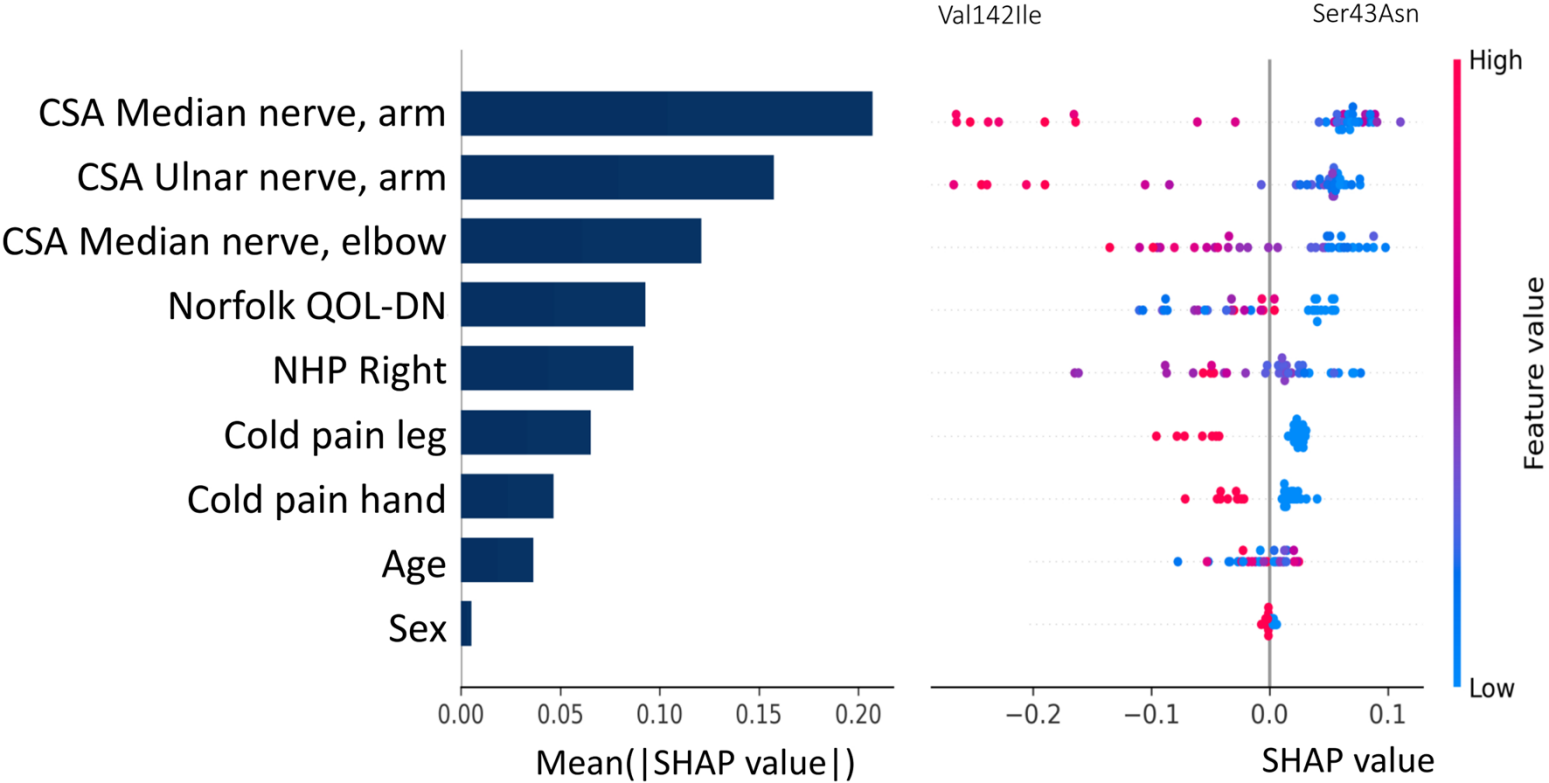
Importance of variables in the predicting mutations using random forest and SHAP, after previous selection. The left side represents the relative importance of the variable, expressed in terms of the Mean(|Shap|value). The right side of each point represents the SHAP value (contribution to the prediction), the further away from 0, the higher the contribution is considered. The colour represents the value of the variable to each prediction, towards red the value of a continuous variable is higher, in a categorical variable it is the value assigned to class 1. For sex: female = 0, male = 1. In the quantitative sensory test: Hyposensitivity = 1, Normal = 0. SHAP: SHapley Additive exPlanations, NHP: Nine-hole peg test, QOL-DN: Quality of Life-Diabetic Neuropathy, CSA: Cross-sectional Area

The final model, which included the variables that contributed most to each prediction in each model, demonstrated that the cross-sectional area of the median and ulnar nerves at the arm and the median nerve at the elbow were the variables that would most likely differentiated individuals with the Val142Ile or Ser43Asn mutations (mean(|SHAP value|) = 0.20, 0.16, 0.12; Fig. 2, left side). Individuals with the Val142Ile mutation exhibited a larger cross-sectional area compared to those with the Ser43Asn mutation (Fig. 2, left side). Individuals with low Norflok QOL-DN scores were more likely to be in the Ser43Asn group, while those with longer times to complete the NHP with the right hand were more likely to be in the Val142Ile mutation group.

### Sensitivity results

The analysis of the models, which included a continuous variable and a categorical variable randomly distributed between the two populations, demonstrated results that were consistent with those of the main analysis (Fig. S1). This analysis established a contribution threshold above chance, with which the same differential variables were identified as in the main analysis, with the exception of the detection of pain due to exposure to cold in the hand and leg. The latter two categorical variables showed a smaller contribution to the predictions than the continuous random variable. However, they showed a greater contribution with the categorical random variable.

### Post-hoc results

Replication of the main analyses stratified by sex showed that the associations between each variable and mutation type were consistent with main results (Fig. S2). However, NHP proved to be a more significant predictor of mutation type in males than in females. CSA of nerves remained robust predictors of mutation.

## Discussion

This cross-sectional study, conducted on 35 participants diagnosed with hATTR-PN, identified potential differentiating clinical markers between carriers of Val142Ile and Ser43Asn mutations. The cross-sectional area of the median and ulnar nerves at the arm and the median nerve at the elbow, as assessed by ultrasound, emerged as significant differentiating variables. These areas may be larger in Val142Ile mutation carriers compared to Ser43Asn carriers. Our results also suggest that Val142Ile mutation carriers may exhibit lower right upper limb motor performance and a higher probability of hyposensitivity to cold pain in the hand and leg. In contrast, Ser43Asn mutation carriers manifest lower levels of quality of life compared to those with Val142Ile.

Our findings suggest that carriers of Val142Ile and Ser43Asn mutations may present clinically distinct phenotypes in patients with hATTR-PN. Previous studies have also described clinical differences between the different mutations, mainly at the cardiac level [30]. Additionally, variations in the prevalence of neuropathies have been reported according to endemic and non-endemic mutations [31]. In Latin America, differences in the prevalence of peripheral neuropathies have also been identified among the mutations present in Brazil, despite the potential low genetic heterogeneity [32].

One of the clinical differences suggested by our results is that abnormalities in QST thermal modalities are more prevalent in the Val142Ile mutation than in the Ser43Asn mutation. This finding is consistent with a previous study in which assessment of the sensitivity of these modalities was used to detect subclinical neuropathies in presymptomatic subjects with late-onset hATTR mutations [26]. In this study, we observed a predominant impact on Aδ fibers over C fibers. Furthermore, another study also reported significant dysfunction of small fibers in patients with early stage hATTR [33]. Consequently, the inclusion of tactile measures could provide further insight into the involvement of large fibers and the primary susceptibility of small fibers to early pathological changes in hATTR. Additionally, it could suggest ways to refine QST protocols to improve the early detection of neuropathic changes in hATTR.

In order to provide a more comprehensive view of patient outcomes, functional assessments such as the NIS, NHP, TUG, SST5, and MSB were included in this study. The results indicated that patients with the Ser43Asn mutation may exhibit a lower level of quality of life than those with the Val142Ile mutation, but with better upper limb motor performance. Previous studies have suggested that genetic variants of the *TTR* gene, such as Val142Ile and other common mutations, have a similar impact on neuropathic progression and quality of life measures as those observed in our study [34]. These findings underscore the importance of assessing motor function, coordination, and balance, which are crucial to patient independence and quality of life. Furthermore, the inclusion of these assessments offers valuable insights into functional abilities and daily living challenges. The value of including some of these scales in the characterisation and follow-up of patients with hATTR has already been demonstrated in previous studies [34–38].

The sensory and motor differences observed between Val142Ile and Ser43Asn mutation carriers may be attributable to underlying pathophysiological mechanisms affecting nerve function and integrity. Specifically, the Val142Ile mutation, associated with an increased propensity for nerve enlargement and sensory dysfunction, may lead to progressive amyloid deposition around the peripheral nerves. This accumulation is likely to compress and damage nerve fibres, particularly small Aδ fibres responsible for thermal and pain sensations. Conversely, Ser43Asn mutation carriers may experience less severe amyloid deposition around peripheral nerves, resulting in less pronounced changes in nerve structure and function, which could contribute to relatively preserved motor function. Differences in nerve CSA may also result from differential amyloidogenicity or variations in fibril structure in amyloid deposits produced by each mutation, resulting in distinct levels of nerve inflammation and degeneration. This finding aligns with previous studies reporting increased deposition around specific nerve fibres as a primary factor in peripheral nerve dysfunction [26, 33].

Our findings on the use of neuromuscular ultrasound in hATTR align with and extend recent research. We observed significant peripheral nerve thickening, especially at proximal superior limb segments. This observation is consistent with other studies that identified multifocal ultrasound abnormalities in patients with hATTR, especially in the ulnar and median nerves at the elbow and axilla [39, 40]. Our study supports the use of ultrasound as a valuable tool for early diagnosis and follow-up of hATTR, providing a comprehensive assessment of nerve involvement that is critical for patient management. The inclusion of a wide range of nerve locations in ultrasound evaluations is crucial to capture the full spectrum of nerve abnormalities in patients with hATTR. The absence of significant changes in CSA in asymptomatic carriers emphasises the posible usefulness of ultrasound in distinguishing between symptomatic and asymptomatic individuals [40]. In addition, a sex-stratified analysis was conducted due to the possibility of an association between CSA and sex. While the results demonstrated consistency in the form of the association, it is not possible to exclude the possibility of an association between sex and the type of association, or of an interaction between CSA and sex. Although no significant association was observed between tibial nerve CSA at the knee and ankle levels after FDR correction—likely due to insufficient statistical power—a potential association was observed in main results. This may represent a false negative, and future studies with larger sample sizes may clarify this association.

While our study provides valuable insights, several limitations must be acknowledged. Firstly, the cross-sectional design limits our ability to infer causal relationships between the observed neurophysiological and ultrasound findings and the clinical manifestations of hATTR. A longitudinal approach would be more appropriate to determine the progression and potential causal links of these findings. Consequently, the findings presented here are intended to expand the existing knowledge about the clinical manifestations of these mutations. However, interpretations regarding disease progression or response to treatment should be made with caution. Secondly, the relatively small sample size, especially within the mutation subgroups, may reduce the generalizability of the results. This limitation is compounded by the genetic homogeneity of the study population, primarily comprised of individuals from Colombia and Ecuador, which may not represent the broader hATTR population. Additionally, the reliance on nonparametric statistical methods, while appropriate for the sample size, may limit the generabilty of the findings compared to parametric approaches in larger cohorts. Another limitation is the potential variability in the ultrasound assessment of nerve cross-sectional areas, influenced by operator technique and experience. Although measures were taken to standardize the procedure, inter-operator variability cannot be entirely excluded. Furthermore, the exclusion of patients with incomplete data may introduce selection bias, potentially skewing the results. Finally, while our study included a comprehensive set of functional and sensory evaluations, other relevant assessments, such as detailed cognitive assessments, environmental factors, health behaviors and lifestyle, as well as complementary Patient-Reported Outcomes, were not incorporated although these factors have been shown to be associated with the progression of other neurodegenerative diseases [41]. Future studies should address these limitations by including larger, more diverse populations, employing longitudinal designs, and expanding the range of clinical assessments to provide a more holistic understanding of hATTR.

## Conclusion

In conclusion, our study suggests that increased nerve cross-sectional areas, quality of life measures, as well as upper limb motor function and sensitivity in different modalities may be possible differentiating factors between Val142Ile and Ser43Asn mutation carriers. Furthermore, these markers could serve as indicators of disease severity and progression, offering potential tools of diagnosis, monitoring and personalized therapeutic intervention for patients with hATTR, improving the quality of life of affected individuals.

## Supplementary Information

Additional file 1.

## Data Availability

The data that support the findings of this study are available from the authors, but restrictions apply to the availability of these data, which were used under the approval and supervision of the CIFEL ethics committee (file number 20240605-001) for the current study, and so are not publicly available. Data are, however, available from the authors upon reasonable request and with permission from the ethics committee of Centro de Investigación en Fisiatría y Electrodiagnóstico and under the respect of anonymization and approval of the study participants.

## Abbreviations

hATTR: Hereditary transthyretin amyloidosis
TTR: Transthyretin
hATTR-PN: hATTR-polyneuropathy
NSI: Neurological impairment scale
QOL-DN: Norfolk quality of life-diabetic neuropathy
Compass-31: Composite Autonomic Symptom Score-31
NHP: Nine-hole peg test
TUG: Timed up and go test
SST5: Sit and stand 5 times test
MSB: Monopodal static balance test
QST: Quantitative sensory testing
NPS: Neuropathic pain scale
SHAP: SHapley Additive exPlanations
CSA: Cross-sectional area
