# Supplementary material for: Clinical differential factors in patients with hereditary transthyretin amyloidosis with Val142Ile and Ser43Asn mutations
Source: Orphanet J Rare Dis. 2024 Dec 20;19:474. doi: 10.1186/s13023-024-03496-0 (PMC11662437; doi:10.1186/s13023-024-03496-0)
Supplement: Supplementary file 1 — Additional file 1. [file 13023_2024_3496_MOESM1_ESM.docx]

Supplementary Data

*Supplementary methods*

To interpret the classification models based on random forests, SHAP values (SHapley Additive exPlanations) were estimated. These values decompose each prediction made by the model into contributions from each variable, allowing a clear understanding of how each factor influences the prediction for an individual. The calculation of SHAP values is grounded in cooperative game theory, where each variable is treated as a "player" in a cooperative game. The SHAP value for a variable represents the average contribution that variable makes to the model’s output across all possible combinations of variables being included or excluded in the model.

The TreeSHAP algorithm was used to calculate these values in this study, taking advantage of the hierarchical structure of decision trees. For each variable, TreeSHAP evaluates the expected change in the model's prediction when that variable is added to subsets of other variables already considered. For example, when assessing the impact of "sex" (male or female) on the likelihood of a given mutation, the model calculates how the inclusion of "sex" changes the prediction for a specific individual compared to the prediction made without this variable. These marginal contributions are averaged over all possible combinations of variables, providing a comprehensive measure of the role "sex" plays in the model’s decisions.

To illustrate, if "sex" has a positive SHAP value of 0.12 for an individual prediction, it means being male (coded as 1 in the model) increases the probability of a specific mutation compared to being female (coded as 0). Conversely, if the SHAP value is negative, it suggests that being male reduces the likelihood of that mutation. This individual-level explanation is complemented by calculating the mean absolute SHAP value (|SHAP value|) across all observations in the dataset, which quantifies the overall importance of "sex" in the model. The mean|SHAP value| is presented visually in bar plots, showing the relative importance of each variable in predicting outcomes. For example, if "sex" has a mean absolute SHAP value of 0.10 and another variable, such as "age," has a value of 0.20, it indicates that "age" is, on average, twice as influential as "sex" in determining the prediction.

**Figure S1. Relative importance of each variable in the prediction of the Val142Ile or Ser43Asn mutation based on the random forest classifier and SHAP values including random distributed categorical and continuous variables.**


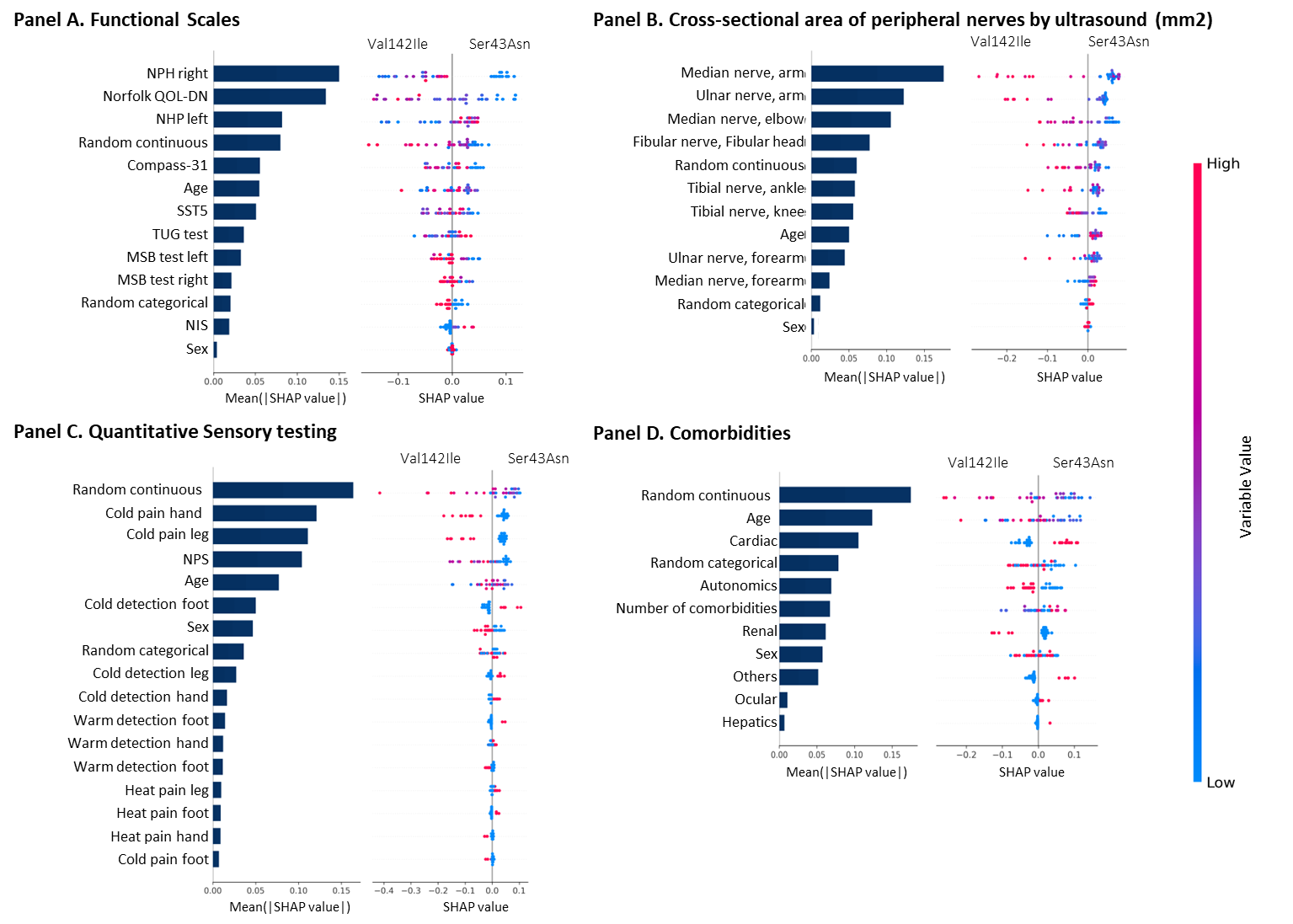


*In each panel, models were adjusted between variables of the same domain, in addition to sex and age. In each panel, the left side represents the relative importance of the variable, expressed in terms of the Mean(|Shap value|). The right side of each point represents the SHAP value (contribution to the prediction), the further away from 0, the higher the contribution is considered. The colour represents the value of the variable to each prediction, towards red the value of a continuous variable is higher, in a categorical variable it is the value assigned to class 1. For sex: female=0, male=1. In the quantitative sensory test: Hyposensitivity=1, Normal=0. In comorbidities: No=0, Yes=1. NSI: Neurological Impairment Scale, TUG: Timed Up and go test, SST5:5-time Sit down-to-Stand up test, NHP: Nine-hole peg test, QOL-DN: Quality of Life-Diabetic Neuropathy, MSB: Monopodal static balance, Compass-31: Composite Autonomic Symptom Score-31, NPS: Neuropathic Pain Scale.*

**Figure S2. Relative importance of each variable in the prediction of the Val142Ile or Ser43Asn mutation based on the random forest classifier and SHAP values stratified by sex.**

*
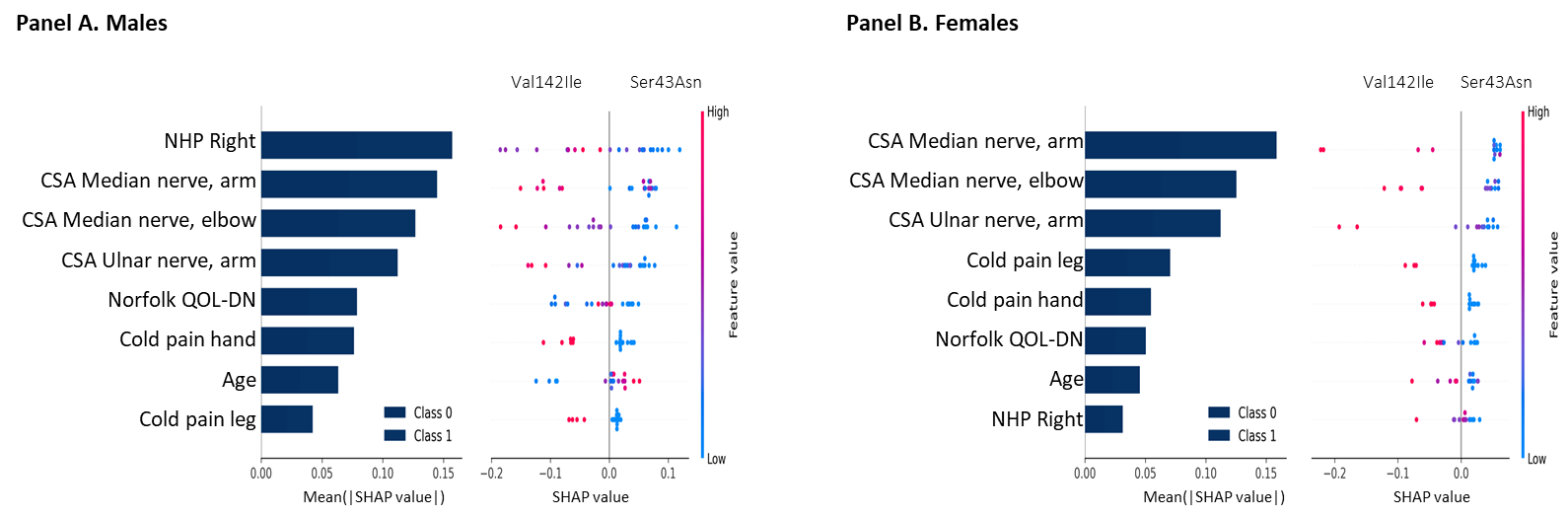
*

*In each panel, the left side represents the relative importance of the variable, expressed in terms of the Mean(|Shap value|). The right side of each point represents the SHAP value (contribution to the prediction), the further away from 0, the higher the contribution is considered. The colour represents the value of the variable to each prediction, towards red the value of a continuous variable is higher, in a categorical variable it is the value assigned to class 1. For sex: female=0, male=1. In the quantitative sensory test: Hyposensitivity=1, Normal=0. In comorbidities: No=0, Yes=1. NSI: Neurological Impairment Scale, TUG: Timed Up and go test, SST5:5-time Sit down-to-Stand up test, NHP: Nine-hole peg test, QOL-DN: Quality of Life-Diabetic Neuropathy, MSB: Monopodal static balance, Compass-31: Composite Autonomic Symptom Score-31, NPS: Neuropathic Pain Scale.*

**Table S1. Detailed description of comorbidities in the analysed population. Number and percentages of participants by condition and mutations. Each participant may have several conditions**

| **Condition** | **Val142Ile** | **Ser43Asn** |
| --- | --- | --- |
| *Cardiac* |  |  |
| Cardiopathy | 2 (20%) | 1 (4%) |
| Heart transplant | 0 (0%) | 3 (12%) |
| Arterial Hypertension | 0 (0%) | 2 (8%) |
| Arrhythmia with Pacemaker | 0 (0%) | 2 (8%) |
| Hypertrophic Cardiomyopathy | 0 (0%) | 1 (4%) |
| Heart Failure | 0 (0%) | 1 (4%) |
| Pericardial Effusion | 0 (0%) | 1 (4%) |
| *Hepatic* |  |  |
| Liver transplant | 0 (0%) | 1 (4%) |
| *Renal* |  |  |
| Urolithiasis | 3 (30%) | 1 (4%) |
| Renal insufficiency | 0 (0%) | 1 (4%) |
| *Autonomic* |  |  |
| Dry eyes | 0 (0%) | 2 (8%) |
| Orthostatic hypotension | 2 (20%) | 8 (32%) |
| Vasomotor Instability | 0 (0%) | 2 (8%) |
| Chronic Diarrhea | 3 (30%) | 2 (8%) |
| Erectile Dysfunction | 0 (0%) | 1 (4%) |
| Cutaneous Discoloration | 0 (0%) | 1 (4%) |
| Chronic Constipation | 0 (0%) | 2 (8%) |
| Hypohidrosis | 1 (10%) | 1 (4%) |
| *Ocular* |  |  |
| Glaucoma | 1 (10%) | 2 (8%) |
| Keratoconus | 0 (0%) | 1 (4%) |
| Periorbital Hyperpigmentation | 0 (0%) | 1 (4%) |
| *Others* |  |  |
| Carpal Tunnel Syndrome | 4 (40%) | 4 (16%) |
| De Quervain’s Tenosynovitis | 0 (0%) | 1 (4%) |
| Rotator Cuff Syndrome | 0 (0%) | 1 (4%) |
| Hypothyroidism | 0 (0%) | 1 (4%) |
| Myopia | 0 (0%) | 1 (4%) |
| Insulin Resistance | 0 (0%) | 1 (4%) |

**Table S2. Contribution of each variable in the model by domains and final model. Estimated contribution by mean absolute Shap value.**

|  | **Mean(\|SHAP value\|)*** | |  |  | **Mean(\|SHAP value\|)*** | |
| --- | --- | --- | --- | --- | --- | --- |
| **Characteristics** | **Model 1** | **Model 2** |  | **Characteristics** | **Model 1** | **Model 2** |
| *Functional Scales* |  |  |  | *Cross-sectional area of the peripheral nerves by ultrasound (mm2)* | | |
| Neurological Impairment Scale | 0.029 | - |  | Median nerve, forearm | 0.029 | - |
| Timed Up and go test | 0.026 | - |  | Median nerve, elbow | 0.129 | 0.121 |
| 5-time Sit down-to-Stand up test | 0.067 | - |  | Median nerve, arm | 0.216 | 0.207 |
| Nine-hole peg test, right | 0.189 | 0.087 |  | Ulnar nerve, forearm | 0.027 | - |
| Nine-hole peg test, left | 0.095 | - |  | Ulnar nerve, arm | 0.151 | 0.158 |
| Norfolk Quality of Life-Diabetic Neuropathy | 0.158 | 0.093 |  | Fibular nerve, Fibular head | 0.075 | - |
| Composite Autonomic Symptom Score-31 | 0.046 | - |  | Tibial nerve, knee | 0.075 | - |
| Monopodal static balance test, right | 0.024 | - |  | Tibial nerve, ankle | 0.087 | - |
| Monopodal static balance test, left | 0.068 | - |  | *Comorbidities* | - |  |
| NPS | 0.104 | - |  | Cardiacs, yes | 0.159 | - |
| *Quantitative Sensory testing, Hyposensitivity* | |  |  | Hepatics, yes | 0.002 | 0.003 |
| Cold detection hand | 0.047 | - |  | Renal, yes | 0.110 | - |
| Cold detection leg | 0.038 | - |  | Ocular, yes | 0.014 | - |
| Cold detection foot | 0.065 | - |  | Otros, yes | 0.059 | - |
| Warm detection hand | 0.026 | - |  | Autonomics, yes | 0.084 | - |
| Warm detection leg | 0.016 | - |  | Number of comorbidities | 0.094 | - |
| Warm detection foot | 0.017 | - |  | Age, Mean (SD) | - | 0.005 |
| Cold pain hand | 0.178 | 0.047 |  | Sex, Female | - | 0.036 |
| Cold pain leg | 0.143 | 0.065 |  |  |  |  |
| Cold pain foot | 0.015 | - |  |  |  |  |
| Heat pain hand | 0.008 | - |  |  |  |  |
| Heat pain leg | 0.015 | - |  |  |  |  |
| Heat pain foot | 0.017 | - |  |  |  |  |

**(Mean(|SHAP value|)): mean absolute Shap value. Model 1: model grouping variables such as functional scales, sensory measures, the cross-sectional area (CSA) by ultrasound, and comorbidities adjusted for age and sex. Model 2: model including the most contributory variables from each model 1 and adjusted for age and sex.*
